# Bibliometric review of primary effusion lymphoma research: Current status, hotspots, and future prospects

**DOI:** 10.1097/MD.0000000000046854

**Published:** 2026-01-09

**Authors:** Yongyu Chen, Bin Liang, Xue Qiu, Peng Cheng, Zhongming Zhang, Ruilin He, Jianchao Ma

**Affiliations:** a Department of Hematology, Minzu Hospital of Guangxi Medical University, Nanning, China; b Department of Gastroenterology, Minzu Hospital of Guangxi Medical University, Nanning, China; c Department of Cardiology, The first Affiliated Hospital of Guangxi Medical University, Nanning, China; d Department of Hematology, The First Affiliated Hospital of Guangxi Medical University, Nanning, China; e Department of Pain Medicine, The Second Affiliated Hospital of Guangxi Medical University, Guangxi, Nanning, China; f Guangxi Medical University, Nanning, China.

**Keywords:** bibliometrics, clinical research, interdisciplinary collaboration, primary effusion lymphoma, viral mechanisms

## Abstract

**Backgrounds:**

Primary effusion lymphoma (PEL) is a rare, highly aggressive malignancy that severely affects patient survival. This study aims to help scholars build a comprehensive framework to identify current challenges, opportunities, and development trends.

**Methods:**

A total of 2544 relevant articles were retrieved from the Web of Science, PubMed, and Scopus databases, encompassing the primary research findings and methodologies in this field. The analysis results indicate that while the number of studies has shown growth in certain years, there remains a challenge of concentrated research themes and a lack of clinical application.

**Results:**

The findings revealed that while the United States dominates PEL research, countries in Asia, particularly China and Japan, are also experiencing growth in this domain. Further keyword co-occurrence analysis indicates that current research hotspots in PEL primarily focus on viral mechanisms, immune evasion, and treatment methods. However, clinical treatment-related keywords such as “chemotherapy agents” and “immunotherapy” appear infrequently in the literature, suggesting a significant disconnect between basic research and clinical application.

**Conclusion:**

The study reveals the current situation and trend of the growing PEL research around the world. The findings of this study not only illuminate current trends in PEL research but also emphasize the necessity of continued scholarly inquiry to address the multifaceted challenges faced by patients and healthcare providers alike.

## 1. Introduction

Primary effusion lymphoma (PEL) is a rare subtype of non-Hodgkin lymphoma, distinct in its clinical manifestations, pathological characteristics, and treatment strategies compared to more common lymphoma types. The primary characteristic of PEL is the accumulation of large lymphoma cells within body cavities, leading to the formation of serous exudate, without the presence of solid tumors. This unique pathological presentation makes PEL challenging to diagnose and treat. In recent years, advancements in medical technology and research have significantly improved the diagnosis and treatment methods for PEL.^[1–3]^ These developments have enriched our understanding of the disease. However, due to the rarity and complexity of the disease, many challenges and unanswered questions remain. Therefore, it is of great significance to comprehensively and systematically analyze the research status of PEL and deeply explore its development trends and future directions to promote the sustainable development of this field and improve the level of clinical diagnosis and treatment.

As a scientific research method, bibliometrics can objectively and quantitatively analyze the research status and development trends in a certain field. Through bibliometric methods, we can fully understand the core research content, main research results, research hotspots, and existing problems and deficiencies in the field of PEL. At the same time, we can also predict the future research direction and development trend in the PEL field, providing valuable reference information for clinicians and researchers.

The study aims to make a comprehensive and in-depth analysis of the current research status of PEL through bibliometric methods. We will analyze the distribution of research forces and research hotspots in the field of PEL from the number, sources, authors, and institutions of published literature. Additionally, we will explore the etiology of PEL in depth to provide useful reference and inspiration for clinicians and researchers.

## 2. Materials and methods

### 2.1. Data retrieval and literature screening

This study was a bibliometric analysis based on publicly available literatures, without human/animal experiments. Thus, the Ethics Committee of Minzu Hospital Guangxi Medical University waived the requirement for informed consent. This study conducted literature searches across 3 databases on July 8, 2025. The initial search was performed in the Web of Science Core Database (WoSCC) using the following search formula: TS= (\ “Lymphoma, Primary Effusion\” OR \ “Effusion Lymphoma, Primary\” OR \ “Effusion Lymphomas, Primary\” OR \ “Lymphomas, Primary Effusion\” OR \ “PELs\” OR \ “PEL\”). A total of 2263 articles were retrieved, with only English papers and reviews included. Additionally, 360 articles of other types were excluded from the analysis. Including Retraction (n = 1), Publication with Expressions of Concern (n = 1), Early Access (n = 4), Correction (n = 11), Proceedings Paper (n = 20), Book Chapter (n = 22), Editorial Material (n = 38), Letter (n = 61), Meeting Abstract (n = 177), and non-English (n = 25). Ultimately, 1903 articles published between 1996 and 2025 were retained. We conducted a PubMed database search using the following search terms: (Lymphoma, Primary Effusion [Title/Abstract]) OR (Effusion Lymphoma, Primary [Title/Abstract]) OR (Effusion Lymphomas, Primary [Title/Abstract]) OR (Lymphomas, Primary Effusion [Title/Abstract]) OR (Primary Effusion Lymphomas [Title/Abstract]) OR (Primary Effusion Lymphoma [Title/Abstract]). This yielded 1642 articles. We only included English papers and reviews, excluding 113 articles of other types. Including Retracted publications (n = 1), Published Erratum (n = 3), Letters (n = 46), Books and Documents (n = 7), Clinical Conference (n = 1), Congress (n = 1), Consensus Development Conference (n = 1), Non-English (n = 53), and finally 1529 articles published between 1996 and 2025; Finally, a search was conducted in the Scopus database using the following search terms: TITLE-ABS-KEY (“Lymphoma, Primary Effusion” OR “Effusion Lymphoma, Primary” OR “Effusion Lymphomas, Primary” OR “Lymphomas, Primary Effusion” OR “Primary Effusion Lymphomas” OR “Primary Effusion Lymphoma”). A total of 2214 articles were retrieved. Only English papers and reviews were included, while 339 other types of articles (including Books [n = 1], Retracted Articles [n = 1], Errata [n = 14], Conference Papers [n = 20], Short Reviews [n = 20], Editorials [n = 25], Notes [n = 31], Letters [n = 74], Book Chapters [n = 75], and Non-English Articles [n = 78]) were excluded. Ultimately, 1875 articles published between 1996 and 2025 were retained. After manual screening to remove duplicates and incomplete articles, Web of Science retained 1903 articles, PubMed 294 articles, and Scopus 347 articles, totaling 2544 articles. Specifically, Web of Science exported plain text files with Full Records and Cited References selected, PubMed used Citation Manager for export, and Scopus generated CSV files with all content selected. Following data export, Citespace was employed to process the data and merge the 3 databases. Figure 1 illustrates the detailed workflow for data collection and processing.

**Figure 1.**
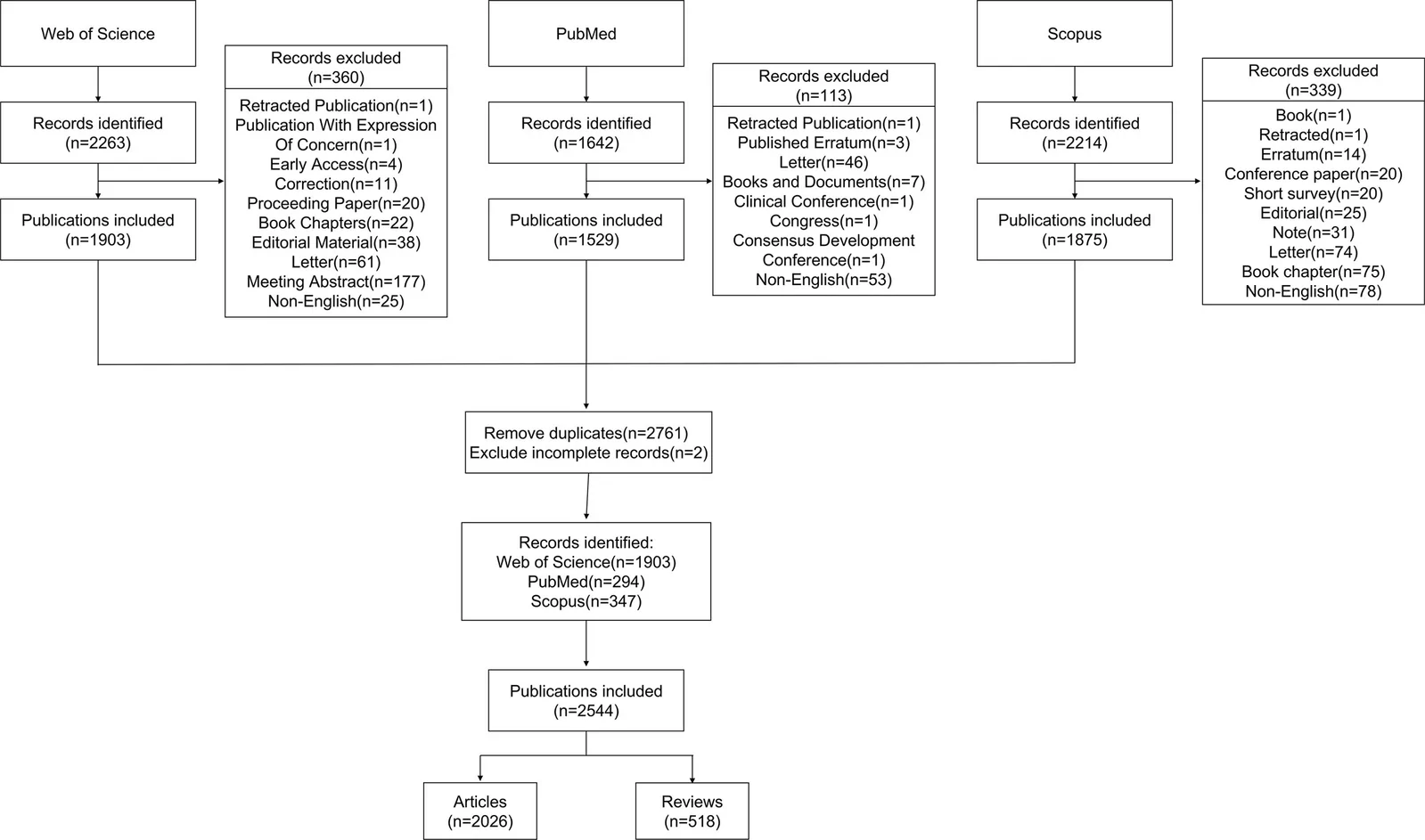
Flow of chart.

### 2.2. Bibliometric analysis

The processed data was analyzed and visualized for bibliometrics in Microsoft Excel 2021, VOSviewer, CiteSpace, and Charticulato.Use Microsoft Excel 2021 to analyze the publication situation from 1996 to 2025 and show the publication trend in this field.

VOSviewer1.6.20 was used to analyze national and author collaborations in collected data, with visualization of the analysis results. Developed by Nees Jan van Eck and Ludo Waltman, VOSviewer^[4]^ (Visualizing Similarities Viewer) is a software tool designed for constructing and visualizing bibliometric networks (Software Survey: VOSviewer, a computer program for bibliometric mapping). Create national collaborative chord diagrams using Charticulator, a Microsoft Research project designed to help researchers customize visual effects (https://donghaoren.org/charticulator/index.html) without code through an online platform.

The analysis employs co-occurrence, clustering, and emergence pattern analysis of CiteSpace6.4. R1 keywords, with visualizations of the results. CiteSpace, a bibliometric visualization tool developed by Professor Chen Chaomei’s team, includes 2 main components: CiteSpace II (Detecting and Visualizing Emerging Trends and Transient Patterns in Scientific Literature) and Science Mapping: A Systematic Review of Literature.

## 3. Results

### 3.1. Publication output and citation trends

The variation in the number of publications within a specific field over time reflects its developmental status and trends. Figure 2 illustrates the annual publication patterns from 1996 to 2025. The data reveals distinct phases of growth: A surge occurred between 1996 and 2001, likely driven by the discovery of HHV-8/KSHV (1994) and its association with PEL etiology, which accelerated both basic and clinical research. From 2002 to 2009, the field entered a plateau period with an average of 85 papers per year, indicating the establishment of core pathogenic mechanisms and diagnostic frameworks. After 2010, it experienced a second peak reaching 122 papers in 2017, reflecting sustained research momentum fueled by advancements in human immunodeficiency virus (HIV) co-infection management, novel diagnostic technologies (e.g., molecular pathology), and therapeutic strategies (including targeted drugs and immunotherapy). Notably, while recent years have seen a decline in paper output, the cumulative total (2544 papers by 2025) demonstrates substantial progress, marking PEL’s evolution from a rare disease to a well-established research field. Linear fitting of cumulative publications (1996-present) shows *R*^2^=0.9955, with the red dashed line representing the fitted curve. This confirms the field’s ongoing growth trajectory and suggests continued expansion. All these results show that this research field is developing and attracting more and more attention.

**Figure 2.**
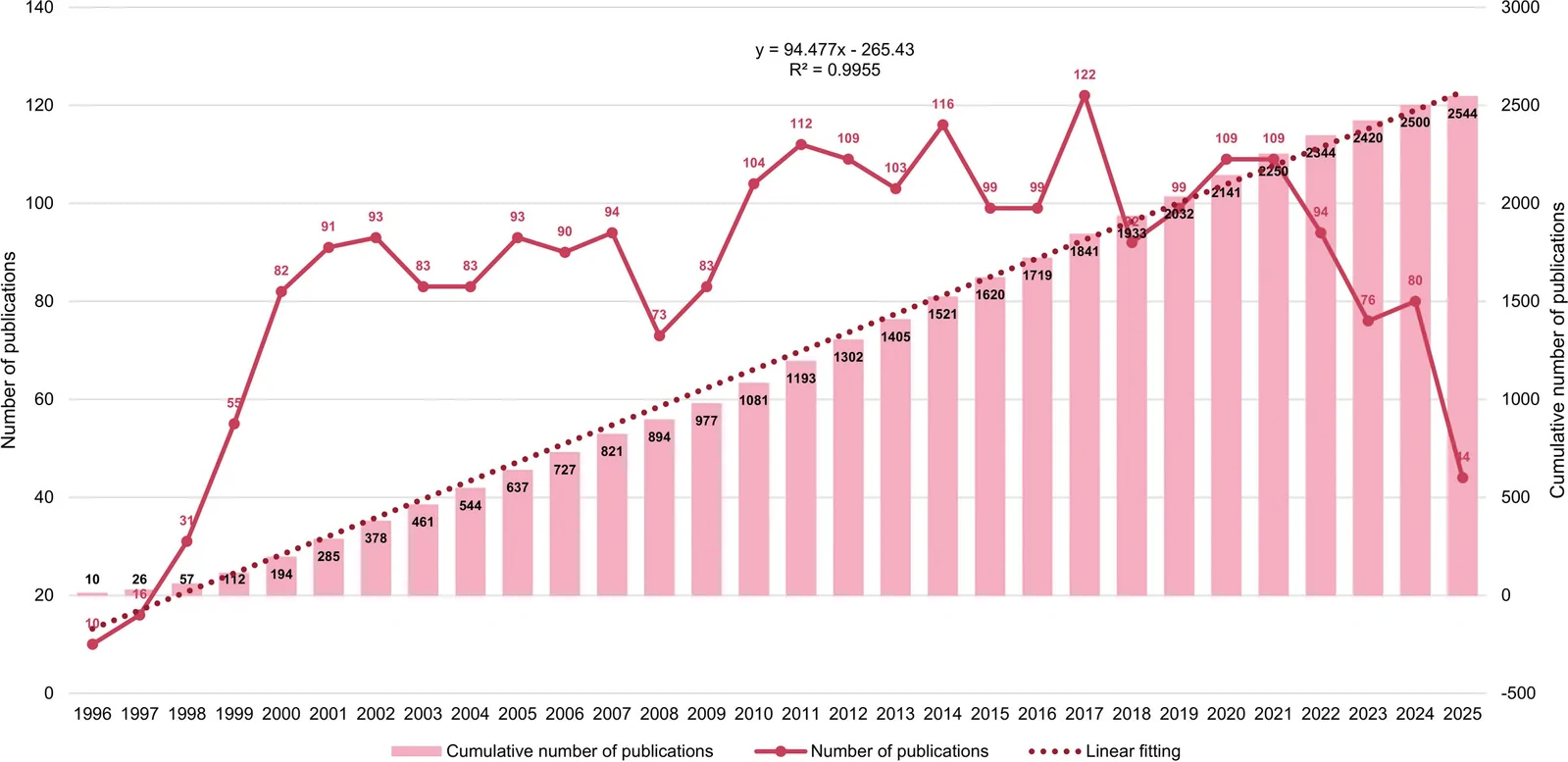
Trends in publication output and citations.

### 3.2. Distribution and cooperation of countries

Over 100 countries worldwide have contributed to this field. Using VOSviewer for international collaboration analysis, we focused on 27 countries with at least 10 publications, as shown in Figure 3A. Node sizes represent publication counts, while connections between nodes indicate cross-national collaborations. The United States dominates with an overwhelming advantage (1353 publications, accounting for over half of core countries), forming the absolute core. Europe has formed an active cluster centered around Italy (243 publications), the UK (172), Germany (138), and France (103). Asia exhibits a regional development pattern marked by intense competition between China and Japan (229 and 222 publications respectively) and South Korea’s (55) catch-up efforts. High-HIV-burden regions like South Africa and Brazil (both with 24 publications) along with resource-rich Saudi Arabia (29 publications) provide unique epidemiological perspectives and regional treatment strategies.

**Figure 3.**
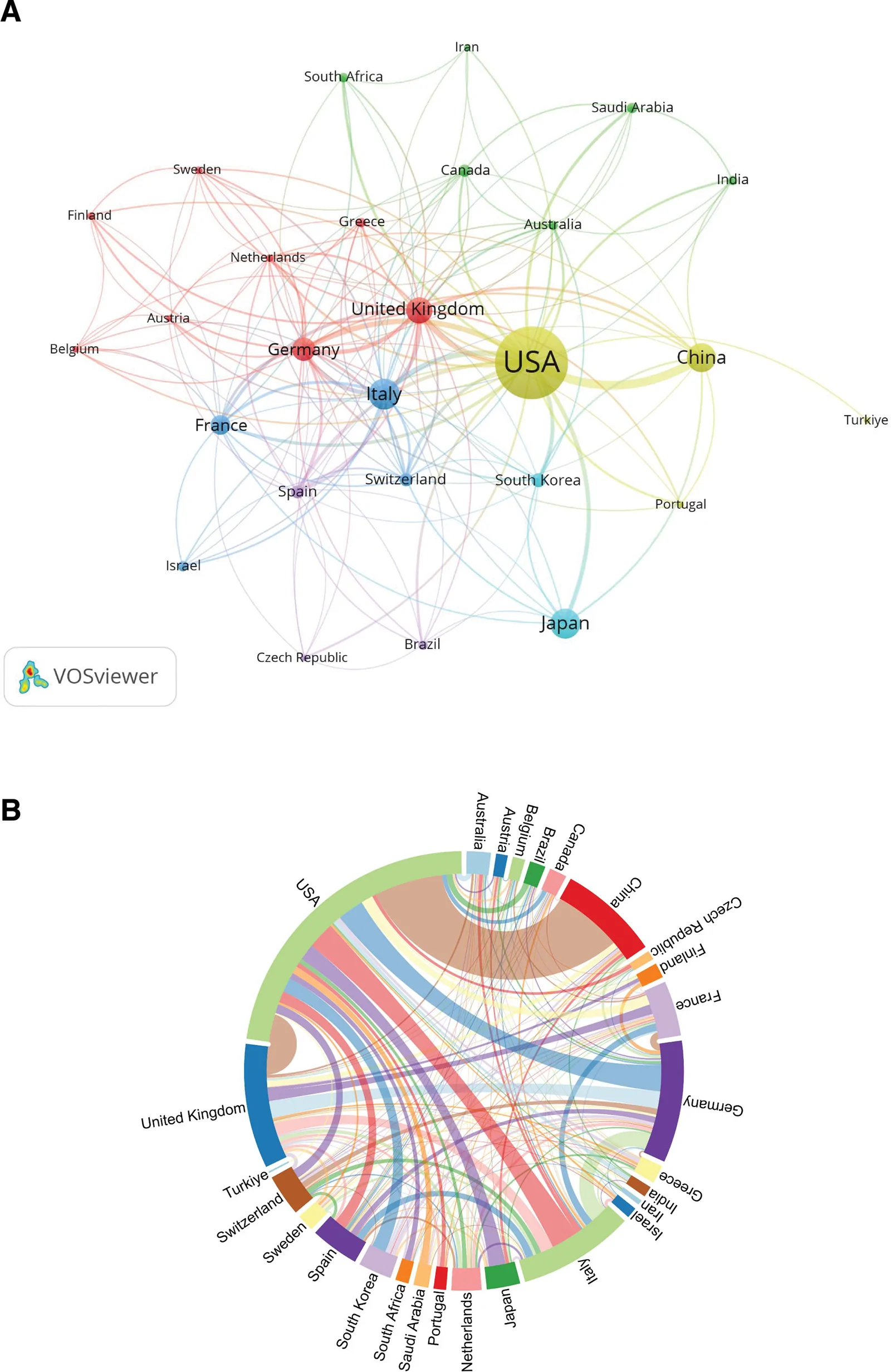
(A) Network of national cooperation; (B) Country cooperation chord diagram.

To better illustrate international cooperation and bilateral collaboration between countries, we created the national cooperation chord diagram in Figure 3B. Node sizes represent total link strength (TLS), indicating the total number of cooperative interactions between countries, while interconnections between nodes denote link strength (LS), reflecting the frequency of collaboration. The United States dominates as a global hub with an overwhelming network hub status (TLS = 365), maintaining its strongest transnational connection with China (LS = 91) and forming deep collaborations with key research partners including Japan (LS = 22), South Korea (LS = 19), and Italy (LS = 36). Europe forms an internal collaboration network centered on Germany and the UK, with Italy serving as a bridge (Germany: LS = 22; France: LS = 10; Spain: LS = 10), though regional cooperation intensity significantly lags behind transcontinental linkages (e.g., Germany–US: LS = 35; UK–US: LS = 37). While China, Japan, and South Korea produce high volumes of publications (229/222/55), their intra-regional connections remain weak – China–Japan (LS = 4) and Japan–Korea (LS = 3) – while Sino–American cooperation accounts for 76% of China’s total linkage strength, highlighting technological dependency. Emerging regions like South Africa (TLS = 16) and Brazil (TLS = 19) contribute clinical resources through medium-intensity links (South Africa–US: LS = 8; Brazil–US: LS = 8), yet retain limited influence in global discourse. On the whole, although this highly centralized network improves efficiency, it may inhibit innovation diversity. In the future, cross-regional equal collaboration should be strengthened to optimize the ecology.

### 3.3. Authors and co-cited authors

A total of 9529 authors participated in research within this field. Using VOSviewer to analyze authors with at least 15 publications (40 individuals), the collaborative network diagram in Figure 4 was generated. The analysis reveals 2 dominant hubs: Carbone Antonino (66 publications, TLS = 103) and Yarchoan Robert (66 publications, TLS = 126), who respectively lead European and North American research communities. Carbone Antonino’s team (Cluster 5) formed a high-density collaboration network in Italy with Gloghini, Annunziata, Gaidano, and others (internal cooperation intensity reaching 46). Meanwhile, Yarchoan Robert’s team (Cluster 1) established a star-shaped radiating clinical research collaboration network with Uldrick, Thomas S., Polizzotto, Mark N., and others (cross-author collaboration frequency exceeding 20 times). Other core teams include the basic research cluster led by Cesarman and Ethel (Cluster 2, characterized by extensive but scattered collaboration), as well as the regional Asian team composed of Cai Qiliang and colleagues (Cluster 4, demonstrating significant internal collaboration but weak international connections). Notably, bridge authors like Bower Mark (who link multiple clusters such as Cluster 1/2/5 simultaneously) contrast with high-productivity researchers like Nicholas John (23 publications with zero collaboration intensity), suggesting the existence of independent research patterns. Emerging teams like Cluster 8 (Cirone, Mara et al) demonstrate the most recent average publication year (2018) and over 33 times of TLS, potentially representing emerging growth points in their respective fields.

**Figure 4.**
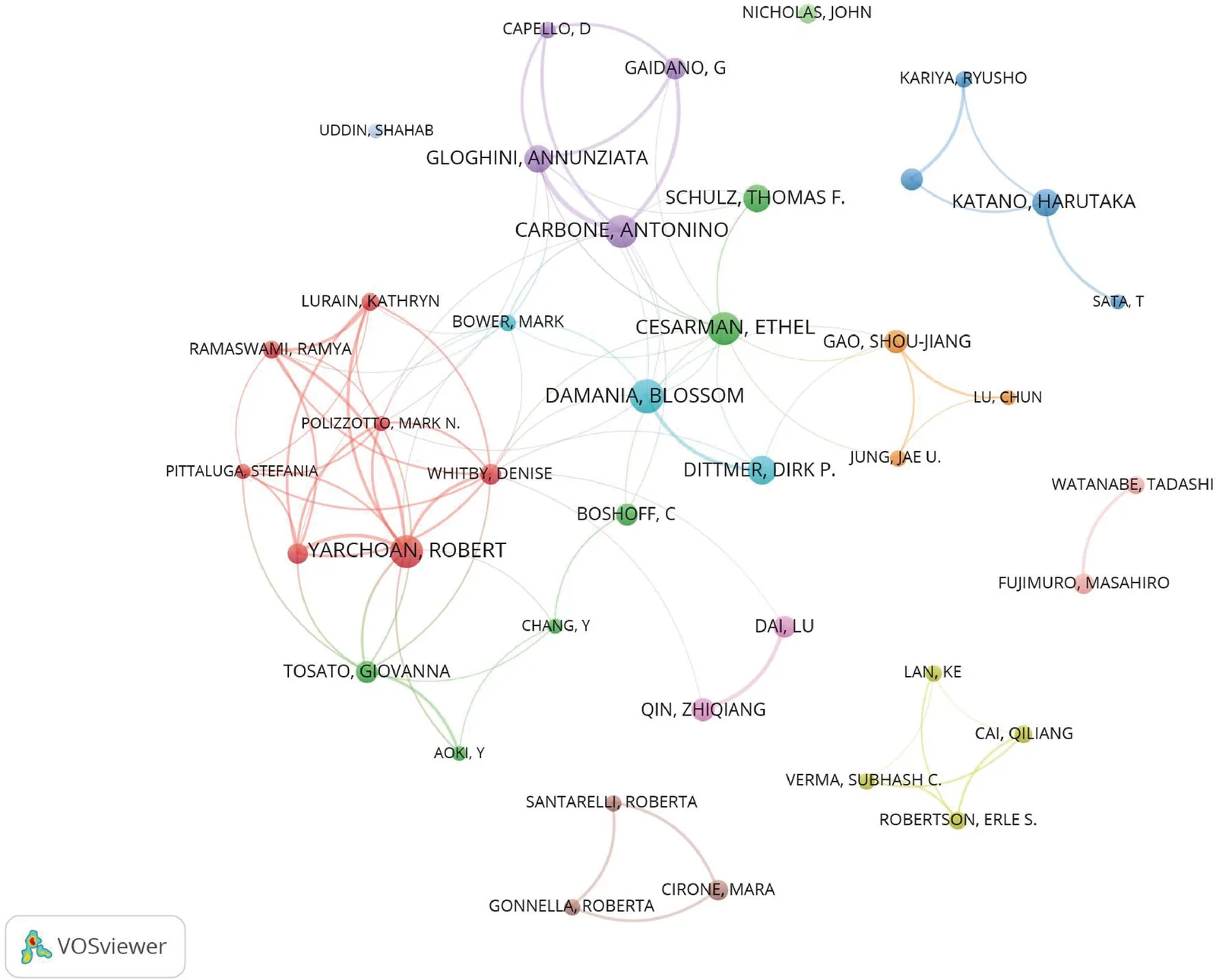
Author collaboration network.

### 3.4. Journals and co-cited journals

Table 1 listed the top 10 journals by publication volume. J Virol (impact factor [IF] = 3.8) from USA leads with 293 articles, followed by BLOOD (IF = 23.1) and PLOS Pathog (IF = 4.9) with 122 and 72 articles respectively. These journals demonstrated strong academic influence and significant contributions to the field. Among the ten journals, BLOOD holds the highest impact factor at IF = 23.1. As the flagship journal of the American Society of Hematology (ASH), BLOOD was first published in 1946 and is recognized as a world-class peer-reviewed weekly in hematology. The journal covers comprehensive research areas including hematopoietic mechanisms, immunotumor biology, thrombosis and hemostasis, and malignant hematologic tumors (including lymphoma), renowned for publishing high-impact original papers and groundbreaking clinical studies. In PEL research, BLOOD holds a unique position: it not only pioneered the landmark discovery linking HHV-8/KSHV virus to PEL etiology but also serves as the preferred platform for disseminating results from current PEL-targeted therapies and immunotherapy clinical trials. Its rigorous peer review standards, fast publication cycle, and extensive academic reach make it a core channel for hematologists and oncologists to access cutting-edge research. Figure 5 illustrated the publication volume and proportion of the top 10 journals.

**Table 1:** Top 10 journals by number of publications.

Rank	Sources	Number of publications	Country	IF (JCR 2024)
1	JOURNAL OF VIROLOGY	293	USA	3.8
2	BLOOD	122	USA	23.1
3	PLOS PATHOGENS	72	USA	4.9
4	VIROLOGY	36	USA	2.4
5	VIRUSES-BASEL	35	Switzerland	3.5
6	LEUKEMIA & LYMPHOMA	34	United Kingdom	2.2
7	CANCERS	28	United Kingdom	4.4
8	JOURNAL OF MEDICAL VIROLOGY	28	USA	4.6
9	ONCOGENE	27	United Kingdom	7.3
10	PLOS ONE	27	USA	2.6

IF = impact factor, JCR = journal citation reports.

**Figure 5.**
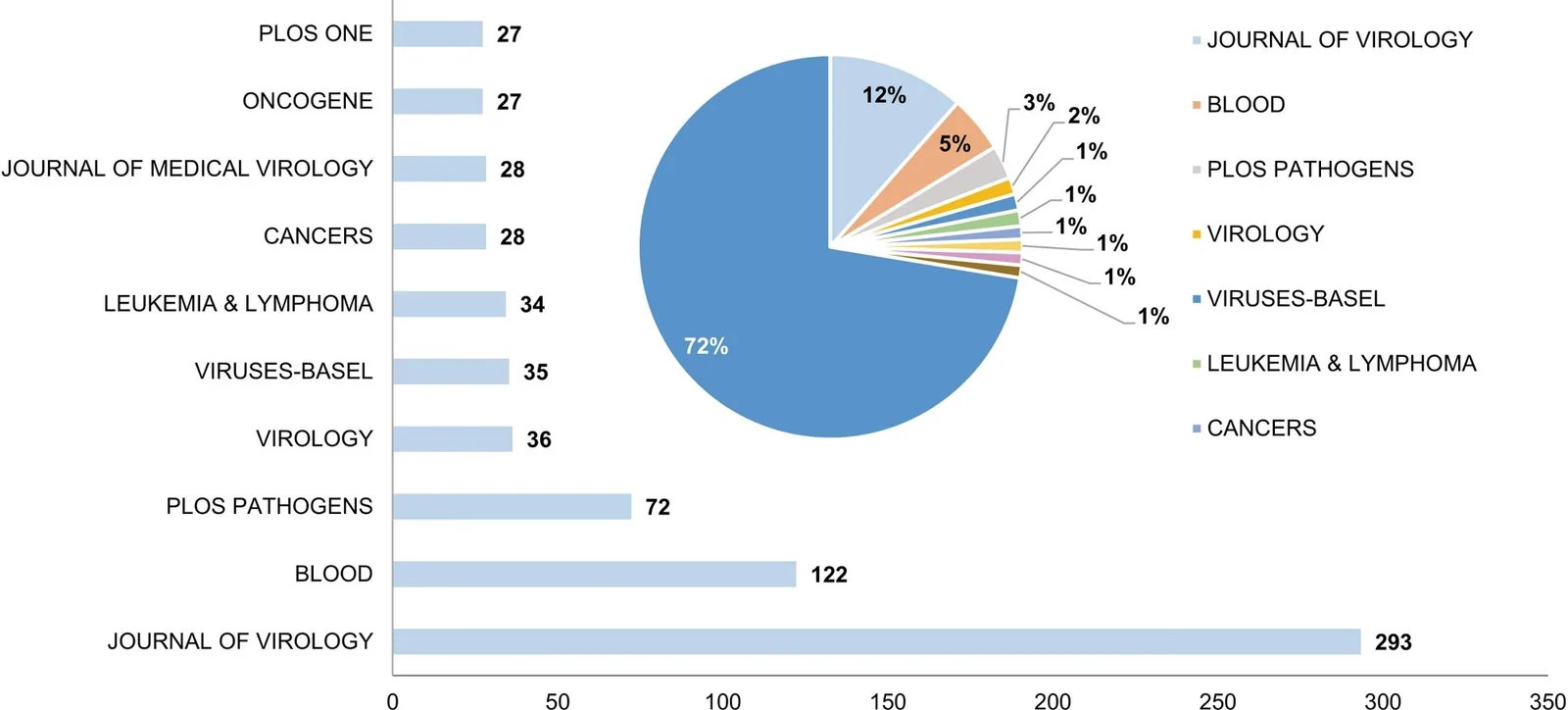
Distribution of journals.

### 3.5. Keyword analysis

This study utilized the g-index to extract 2544 articles, setting *k* = 10 for keyword extraction. A total of 387 keywords were identified through merging and deletion processes. The merged keywords were synonyms, while the deleted ones were deemed irrelevant or nonsignificant. After creating merged and deleted files, the analysis was recalculated by reducing k to 3 to prevent redundant keyword identification. This resulted in 150 unique keywords. The final computational results were visualized to facilitate interpretation.

Figure 6A presents a keyword co-occurrence network diagram where nodes represent keywords and their sizes indicate frequency. Larger nodes correspond to more frequent occurrences. Node colors transition from blue at the center to red at the periphery, reflecting decreasing temporal proximity (as shown in the lower-left legend). Each color corresponds to specific years, with circular bands on nodes showing annual frequency: wider bands indicate higher occurrence. Purple bands around peripheral nodes signify nodes with >0.1 centrality, indicating pivotal bridging roles. Connecting lines between nodes show co-occurrence frequency, with thicker lines denoting higher co-occurrence. Core domains are dominated by viral mechanism keywords, including “kaposis sarcoma” (586 mentions, 0.36 centrality), “Epstein–Barr virus (EPV)” (523 mentions, 0.36), and “herpesvirus sarcomatogenic” (531 mentions, 0.32), forming dense clusters. Pathogen-related concepts like “human herpesvirus 8” (428 mentions) and “kSHV” (335 mentions) are also clustered together. Clinical treatment keywords are severely marginalized, with therapeutic agents like “rituximab” (31 mentions) and “doxorubicin” (13 mentions) accounting for <5% of viral-related keywords, showing a central tendency approaching zero. Notably, high-centrality bridge terms such as “multicentric Castlemans disease” (222, 0.18) and “plasmablastic lymphoma” (102, 0.13), while linking HIV-related immune deficiency (“acquired immunodeficiency syndrome [AIDS]”) and lymphoma subtypes, fail to effectively connect basic research with clinical studies. This creates a fragmented disconnect between viral mechanisms (e.g., “latency”) and clinical terminology clusters (e.g., “diagnosis”). Conversely, low-frequency yet highly central terms like “Hodgkin’s disease” (0.52 centralization) and “metabolism” (0.43) suggest underdeveloped cross-disciplinary hotspots. This structural imbalance reveals fundamental contradictions: While HHV-8/KSHV virology research has accumulated substantial expertise, therapeutic innovations (targeted drugs, immunotherapies) and technological advancements (liquid biopsy, molecular imaging) remain nearly absent. Urgent action is needed to bridge knowledge gaps through pathway studies (e.g., “nf-kappa b” [174 mentions]) and intervention strategies (e.g., “inhibition” [31 mentions]), breaking the unipolar knowledge structure.

**Figure 6.**
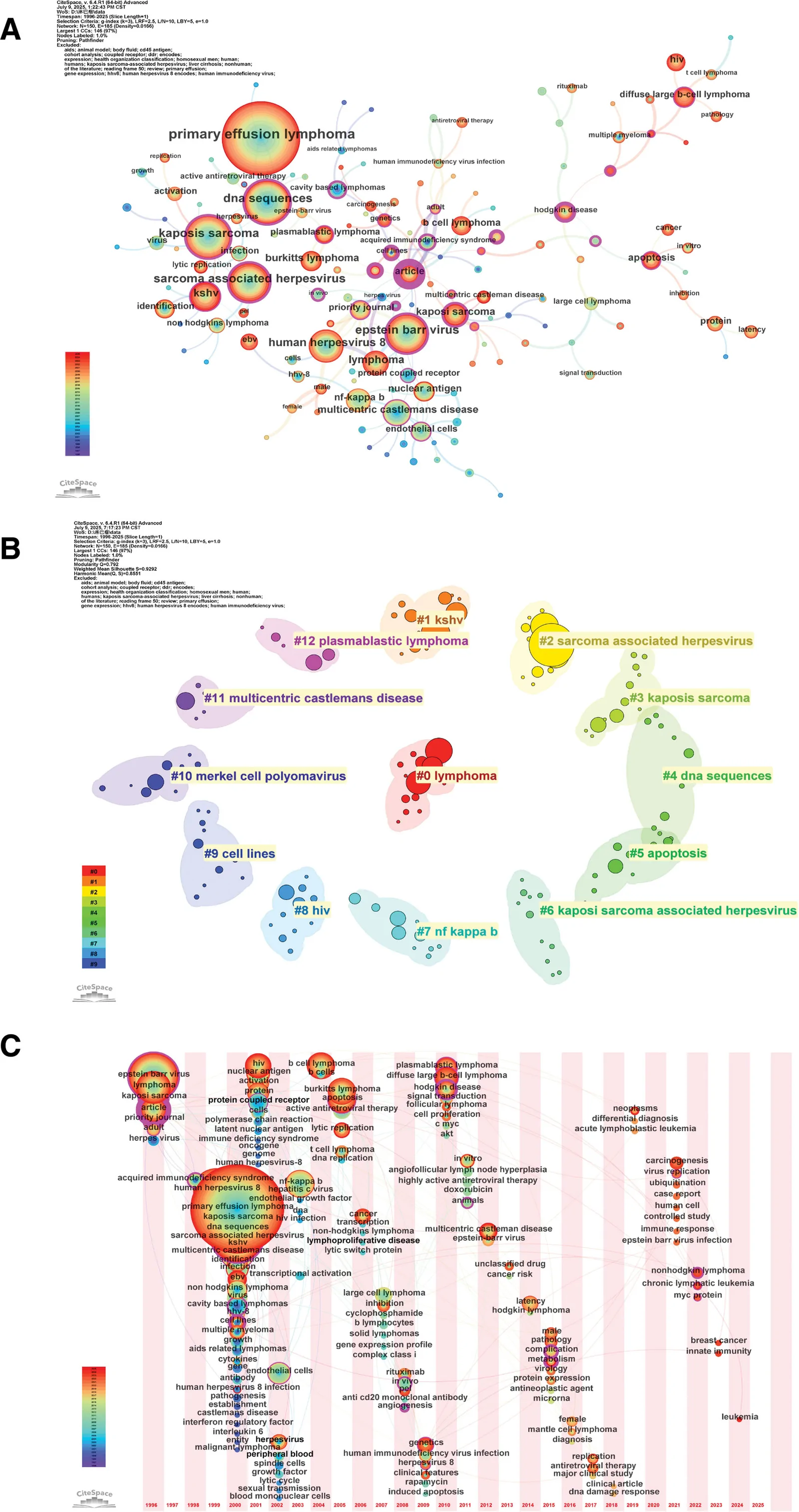
(A) Keyword co-occurrence network; (B) Keyword clustering; and (C) Keyword time zone map.

Figure 6B presents a keyword clustering analysis by utilizing the Citespace log-likelihood ratio (LLR) algorithm to extract cluster labels from keyword fields. Thirteen clusters were identified. The *Q*-value of 0.792 (significantly higher than 0.3) indicates significant clustering structure, while the *S*-value of 0.9292 (exceeding 0.7) demonstrates compelling statistical validity. These clusters correspond to: #0 lymphoma, #1 KSHV, #2 sarcoma-associated herpesvirus, #3 Kaposi sarcoma, #4 DNA sequences, #5 apoptosis, #6 Kaposi sarcoma-associated herpesvirus, #7 NFκB, #8 HIV, #9 cell lines, #10 Merkel cell polyomavirus, #11 multicentric castlemans disease, and #12 plasmablastic lymphoma. These clusters collectively represent distinct research directions within the field.

#### 3.5.1. Cluster 0: basic research and clinical relevance (virus-population-diagnosis)

Centered on “Epstein–Barr Virus” (EBV) and “Human Herpesvirus 8” (HHV-8), this cluster integrates co-infectious factors such as “lymphoma” and “HCV” through “diagnosis” and “case reports,” with clinical observations connected via “case reports.” While reflecting an initial framework linking viral etiology to clinical manifestations, the high-frequency term “priority journals” suggests that most research findings are published in high-impact platforms.

#### 3.5.2. Cluster 1: virus carcinogenesis mechanism (activation and replication of KSHV)

The study focuses on “kaposis sarcoma” (Kaposi sarcoma) and “kshv” (KSHV virus), extending to molecular mechanisms such as “replication” and “DNA damage response,” while linking these to the immunodeficiency background of “HIV infection.” Keywords like “lytic switch protein” highlight the depth of research into viral life cycle regulation.

#### 3.5.3. Cluster 2: molecular basis of virology (genome and pathogenic entity)

Centered on “DNA sequences” and “sarcoma-associated herpesvirus,” this framework encompasses disease entities such as “primary effusion lymphoma” (PEL) and “PEL,” along with functional terms including “lytic replication” and “c-myc” (a cancer gene). These elements collectively form the core knowledge module explaining how viral genomes manipulate host mechanisms.

#### 3.5.4. Cluster 3: clinical management of HIV co-infection

The triangle formed by “AIDS”), “b cell lymphoma” (B cell lymphoma) and “antiretroviral therapy” (antiretroviral therapy) reveals the clinical particularity of PEL under the background of HIV immune deficiency, while the lack of “controlled study” reflects the lack of evidence-based medical evidence.

#### 3.5.5. Cluster 4: traditional therapy and signaling pathways

Chemotherapy and immunotherapy agents such as “doxorubicin” (doxorubicin) and “rituximab” (rituximab) are listed alongside “signal transduction” (signal transduction) and “cell proliferation” (cell proliferation), highlighting the current reliance on conventional treatment regimens with superficial mechanistic studies. The frequent use of the term “complication” (complications) underscores challenges in managing therapeutic side effects.

#### 3.5.6. Cluster 5: apoptosis and targeted intervention

The intersection of “apoptosis,” “inhibition,” “akt” (protein kinase B), and “latency” (latent period) forms an experimental therapeutic cluster targeting the apoptosis pathway. While “unclassified drugs” and “myc protein” indicate potential new targets, their low frequency suggests delayed translational progress.

#### 3.5.7. Cluster 6: mechanism of carcinogenesis and immune interaction

Molecular events such as “carcinogenesis” (carcinogenicity) and “ubiquitination” (ubiquitination) are juxtaposed with “immune response” (immune response) and “microRNA” (microRNA), suggesting the cross-mechanism of viral carcinogenesis and immune escape, while heterogeneous terms such as “breast cancer” (breast cancer) reflect the blurred boundaries of the field.

#### 3.5.8. Cluster 7: viral protein interaction with host

“NFK-kappa B,” “nuclear antigen,” “EBV,” and “HHV-8” focus on the molecular interface of viral proteins that regulate host signaling pathways, and “spindle cells” suggest tumor microstructure association.

#### 3.5.9. Cluster 8: differentiation of lymphoma subtypes

Heterogeneous subtypes such as “diffuse large B-cell lymphoma” (DLBCL) and “follicular lymphoma” (FL) co-occur with “HIV” and “pathology,” highlighting the complexity of PEL’s differential diagnosis, while “multiple myeloma” (MM) is a very uncommon association.

#### 3.5.10. Cluster 9: cellular models and molecular subtypes

“Cell lines” (cell lines) and “AIDS-related lymphoma” (AIDS-related lymphoma) support the experimental system, while “protein expression” (protein expression) and “gene expression profile” (gene expression profile) point to molecular typing attempts. However, vague words such as “entity” (disease entity) reflect the taxonomic dilemma.

#### 3.5.11. Cluster 10: microenvironment and targeting exploration

The most transformative clusters are angiogenesis (blood vessel formation), interleukin-6 (IL-6) connecting the tumor microenvironment, in vivo (in vivo) experiments and rapamycin (RA) marking experimental therapeutics, and multicentric Castleman disease (MCDC) as a key comorbidity bridge.

#### 3.5.12. Cluster 11: immunopathology and detection techniques

“Interferon regulatory factor” (interferon regulatory factor) and “latent nuclear antigen” (latent nuclear antigen) are related to immunopathology, and “polymerase chain reaction” (PCR) is the mainstream detection method. The small cluster size reflects the weak research on immune microenvironment.

#### 3.5.13. Cluster 12: special subtypes and targeted therapy

The terms “plasmablastic lymphoma” (plasma cell lymphoma) and “Burkitt’s lymphoma” (Burkitt lymphoma) emphasize the distinct pathological subtypes. While “anti CD20 monoclonal antibodies” represent a limited number of targeted therapies, the insufficient frequency of “active antiretroviral therapy” highlights the lack of a systematic approach to combination treatment.

The time zone chart of keywords in Figure 6C shows that the horizontal coordinate represents the first occurrence time of keywords. According to the figure, the research in this field can be divided into 3 stages:

#### 3.5.14. First stage: etiological foundation period (1996–2000)

This phase was characterized by the establishment of virological frameworks and disease ontology definitions. The groundbreaking terminology “EBV”, “Kaposi Sarcoma” (Kaposi sarcoma), and “lymphoma” (lymphoma) emerged in 1996, marking the official launch of research in this field. By 2000, pathogen concepts such as “Human Herpesvirus 8” (HHV-8), “herpesvirus-associated sarcoma,” and “Primary Effusion Lymphoma” (PEL) were systematically established. Concurrently, key complications like “Multicentric Castleman Disease” and “AIDS-related Lymphoma” gained prominence, forming an initial tripartite research paradigm of “virus-disease-immune deficiency.” These developments laid the groundwork for subsequent mechanistic exploration.

#### 3.5.15. The second stage: mechanism deepening period (2001–2010)

The research focus shifted toward initial exploration of molecular pathogenic mechanisms and therapeutic pathways: From 2001 to 2005, keywords such as “nf-kappa b” (nuclear factor κB), “apoptosis” (cell death), and “latency” (delay period) revealed the molecular logic of viral host manipulation. Therapeutic terms like “doxorubicin” (2001) and “rituximab” (2008) marked the introduction of traditional chemotherapy/immunotherapy regimens. From 2006 to 2010, advancements in experimental models driven by “in vivo” (in vivo experiments, 2008) and “in vitro” (in vitro experiments, 2011) studies led to emerging subtypes and targets including “plasma cell lymphoma” (2010) and “akt” (aktin-binding domain protein B, 2010). However, targeted therapies like “rapamycin” (2009) remained sporadic explorations, reflecting a significant disconnect between mechanistic depth and therapeutic innovation.

#### 3.5.16. The third stage: transformation exploration period (2011–2024)

The field confronts a stark contradiction between “foundational redundancy” and “clinical scarcity”: On one hand, core concepts like “ubiquitination” (2021) and “DNA damage response” (2018) continue to emerge as key mechanisms, while emerging terms such as “innate immunity” (2023) and “immune response” (2021) hint at nascent research in immune microenvironment studies. Conversely, therapeutic keywords lag significantly behind with fragmented progress. The term “unclassified drugs” (2013) exposes the disorderly development of targeted therapies, while new targets like “myc protein” (2022) only gained attention 2 years later. Notably absent from discussions are hot topics in immunotherapy such as “car-T” and “immune checkpoint,” with diagnostic technologies remaining stuck at “polymerase chain reaction” (PCR, 2001). This reveals fundamental challenges of fragmented translational pathways and technological innovation stagnation within the field.

Keyword prominence analysis aids in exploring shifts in research hotspots within a field, understanding current focal points, and predicting future trends. Table 2 lists the top 25 keywords by prominence value. During the etiology establishment phase (1996–2000), prominence peaks emerged in pathogen studies and disease ontology definitions: “herpes virus” (10.7 intensity, 1996–2003) and “AIDS” (9.53 intensity, 1998–2001) first highlighted the HIV/KSHV co-infection framework. The core concept cluster erupted in 2000, with “multicentric Castleman disease” (28.89 intensity), “cavity-based lymphomas” (21.04 intensity), and “DNA sequences” (17.83 intensity) collectively defining the “virus-complications-genome” triad research paradigm, reflecting concentrated foundational work during this period. In the mechanism deepening phase (2001–2010), focus shifted to technical models and therapeutic exploration: “cell lines” (10.1 intensity, 2000–2002) and “cells” (11.7 intensity, 2001–2009) marked the maturation of in vitro experimental systems. Post-2005 emergence of therapeutic-related terms: “Active antiretroviral therapy” (influence 16.74, 2005–2011) revealed the antiviral treatment boom, while “in vivo” (influence 9.94, 2008–2013) propelled in vivo experimental development. However, mechanism-related terms like “signal transduction” (influence 10.06) only briefly emerged (2010–2011), exposing a temporal mismatch between mechanistic research and technological application, with therapeutic exploration lagging nearly a decade behind etiological studies. During the translational exploration phase, 2 key trends emerged: On one hand, “diffuse large B-cell lymphoma” (influence 29.68, 2017–2025) became the absolute core of this stage with its historically strongest emergence intensity, marking lymphoma subtype differentiation as the new dominant direction. On the other hand, foundational terms like “latency” (influence 18.17, 2014–2020) and “carcinogenesis” (influence 16.7, 2021–2023) regained popularity regarding viral latency and carcinogenic mechanisms, while established terms like “EBV” (influence 14.28, 2021–2025) and “HIV” (influence 9.88, 2022–2025) highlighted renewed innovation fatigue. The sudden disappearance of treatment-related terms (no new therapeutic terms after 2011) and the complete absence of immune microenvironment and modern diagnosis and treatment technology terms in the list of sudden emergences reveal that the field is trapped in a vicious circle of subtype reclassification and repeated mining of mechanisms, and it is urgent to inject external technical concepts to break the deadlock.

**Table 2:**
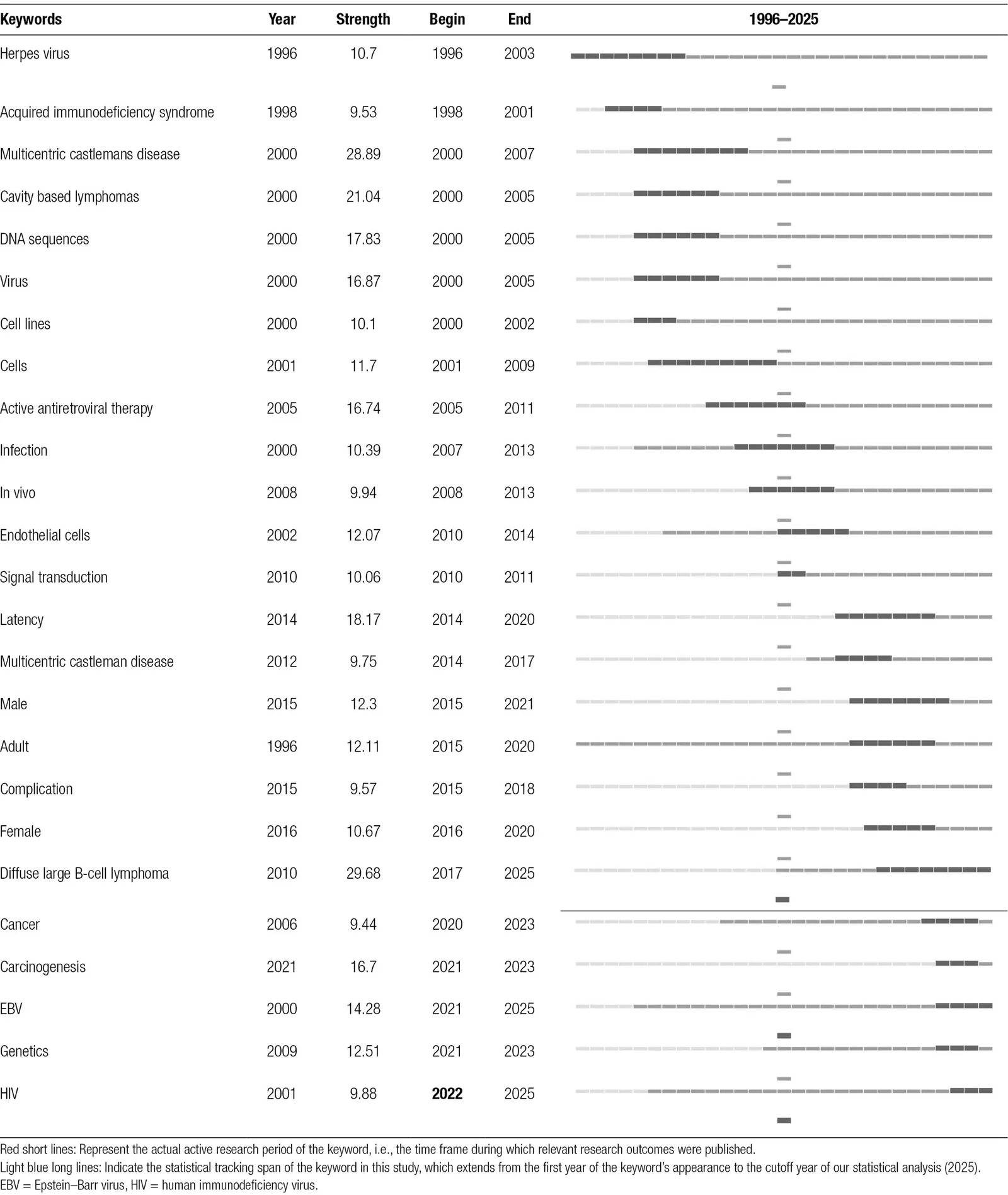
Top 25 keywords with the strongest citation bursts.

## 4. Discussion

The study provides a comprehensive analysis and discussion of the research field of PEL. We analyzed 906 articles published between 1996 and 2025 across various journals, authors, and countries, illustrating the field’s development. Overall, PEL research has shown a trend of increasing year by year. In recent years, advancements in medical technology and deeper research have led to a rapid increase in literature on PEL, demonstrating the field’s ongoing vitality.

Research on PEL represents a critical advancement in the understanding of this rare and aggressive type of lymphoma associated with human herpesvirus 8 (HHV-8). Our study fills significant gaps in the existing literature by providing a comprehensive bibliometric analysis that encapsulates epidemiological trends and collaborative networks in PEL research over the past 2 decades.^[5–7]^ Unlike previous studies that primarily focused on the clinical aspects or treatment outcomes of PEL, our work highlights the surge in research activity since 2010, reflecting an increased global focus on understanding the complexities of this malignancy and its clinical management. For instance, we found that a notable portion of recent publications has explored novel therapeutic targets linked to the oncogenic mechanisms of HHV-8, which were previously under-investigated in human models.^[8]^ This contributes to a broader understanding of how HHV-8 drives lymphomagenesis in PEL, thereby paving the way for future research that can lead to more effective treatments and improved patient outcomes.

The implications of our findings extend beyond academic interest; they hold substantial potential for influencing clinical practice and policy-making. The identification of significant patient characteristics, such as the high prevalence of HIV co-infection among PEL patients, underscores the need for tailored screening and treatment approaches. Furthermore, the superior response rates associated with combination therapies involving Rituximab highlight the necessity for clinical guidelines that prioritize such strategies in managing PEL.^[9,10]^ These insights can inform healthcare policies aimed at optimizing resource allocation and enhancing treatment protocols for this vulnerable patient population, ultimately improving prognosis and survival rates.

Based on the current research status and development trend in the field of PEL, we believe that the future research direction mainly includes the following aspects: First, strengthening basic research to deeply explore the pathogenesis and etiology of PEL. Understanding the pathogenesis and etiology of PEL will enable us to develop more effective strategies for prevention and treatment in clinical settings. Second, optimizing diagnostic techniques and methods to improve the diagnostic accuracy and efficiency of PEL. As science and technology advance, we must explore and adopt new diagnostic techniques to meet clinical needs. Third, exploring more effective treatment strategies and programs. Given the limited effectiveness of single chemotherapy regimens, we should enhance our research on combination treatments and individualized strategies. Fourth, strengthening the study of prognostic factors and survival analysis. By analyzing the factors that affect the prognosis of PEL patients, we can offer clinicians more accurate prognostic assessments. This will help guide clinical treatment and improve patient outcomes. In addition, with the continuous development of precision medicine, we also need to strengthen research in genomics, proteomics, and other areas to reveal the molecular biological characteristics and individual differences of PEL. This will help us achieve more accurate diagnosis and treatment, improving the quality of life and survival rates of patients. At the same time, interdisciplinary cooperation and exchange is also an important direction for the future development of PEL. By collaborating with disciplines like immunology and virology, we can explore the pathogenesis and treatment of PEL from various perspectives, revitalizing the field’s development. Furthermore, strengthening international cooperation and exchanges is crucial for promoting the development of the PEL field. Through collaboration and communication with international research institutions and teams, we can share resources, technology, and experience to jointly advance the progress of the PEL field. Finally, we must also focus on social and ethical issues in the PEL field. For example, how to protect patient privacy and how to reasonably allocate medical resources are issues that require deep consideration and discussion.

However, it is vital to acknowledge the limitations inherent in our study. While our bibliometric analysis provides valuable insights into publication trends and collaborative networks, it does not encompass clinical validation or real-world outcomes, which are essential for translating research findings into practice. Additionally, the retrospective nature of the literature review may introduce biases, and the exclusion of non-English publications could limit the reliability of our conclusions. Future studies should focus on longitudinal analyses that integrate clinical data with bibliometric insights to provide a more comprehensive understanding of PEL, including the long-term efficacy and safety of emerging therapies by addressing these limitations, subsequent research can further refine prognostic models and therapeutic strategies, ultimately enhancing the management of PEL and improving patient outcomes.

The limitations of this study should be acknowledged, as they may impact the interpretation of the findings. Primarily, the lack of clinical validation analysis restricts our ability to generalize the results to real-world patient populations, potentially undermining the applicability of the identified trends. Additionally, the retrospective nature of the literature review may introduce inherent biases, as the studies included may vary in methodology, sample size, and reporting quality. Furthermore, the exclusion of non-English publications may limit the comprehensiveness of our bibliometric analysis, potentially overlooking valuable insights from diverse research contexts. These factors collectively suggest that while our study provides a significant overview of PEL research, further investigation is warranted to validate and expand upon these findings in a clinical setting.

## 5. Conclusion

In conclusion, this study underscores the critical advancements in the understanding of PEL and highlights essential areas for future exploration. The insights gained regarding patient characteristics, treatment regimens, drug efficacy, side effects, and prognostic evaluation position this research as a foundational contribution to ongoing efforts aimed at improving patient outcomes in this challenging disease. As the field continues to evolve, it is imperative that researchers pursue innovative therapeutic approaches and further investigate the identified biomarkers to enhance clinical management strategies. The findings of this study not only illuminate current trends in PEL research but also emphasize the necessity of continued scholarly inquiry to address the multifaceted challenges faced by patients and healthcare providers alike.

## Acknowledgments

The authors thank the developers of VOSviewer, CiteSpace, and another bibliometric tool.

## Author contributions

**Investigation:** Bin Liang, Jianchao Ma.

**Methodology:** Yongyu Chen, Zhongming Zhang, Jianchao Ma.

**Project administration:** Yongyu Chen, Zhongming Zhang.

**Resources:** Xue Qiu, Zhongming Zhang, Jianchao Ma.

**Software:** Bin Liang, Xue Qiu, Peng Cheng, Ruilin He.

**Supervision:** Peng Cheng, Ruilin He, Jianchao Ma.

**Validation:** Yongyu Chen, Bin Liang, Xue Qiu, Ruilin He.

**Visualization:** Yongyu Chen, Xue Qiu, Ruilin He.

**Writing – original draft:** Yongyu Chen, Bin Liang.

**Writing – review & editing:** Peng Cheng, Ruilin He, Jianchao Ma.

## Supplementary Material
